# An approximation of one-dimensional nonlinear Kortweg de Vries equation of order nine

**DOI:** 10.1371/journal.pone.0262157

**Published:** 2022-01-07

**Authors:** Sidra Saleem, Malik Zawwar Hussain, Imran Aziz

**Affiliations:** 1 Department of Mathematics, Lahore College for Women University, Lahore, Pakistan; 2 Department of Mathematics, University of the Punjab, Lahore, Pakistan; 3 Department of Mathematics, University of Peshawar, Peshawar, Pakistan; China University of Mining and Technology, CHINA

## Abstract

This research presents the approximate solution of nonlinear Korteweg-de Vries equation of order nine by a hybrid staggered one-dimensional Haar wavelet collocation method. In literature, the underlying equation is derived by generalizing the bilinear form of the standard nonlinear KdV equation. The highest order derivative is approximated by Haar series, whereas the lower order derivatives are attained by integration formula introduced by Chen and Hsiao in 1997. The findings are shown in the form of tables and a figure, demonstrating the proposed technique’s convergence, robustness, and ease of application in a small number of collocation points.

## 1 Introduction

Many problems that have arisen in different eras of science and engineering have been described using linear and nonlinear phenomena. While some of these issues can be solved immediately, a considerable number of them remain at the cutting edge of mathematical modelling. As a result, partial differential equations (PDEs) have become an important tool for describing such processes. A clear understanding of modelling is essential to deal with such situations [1]. The authors were driven to find analytic, semi analytic, or numerical solutions to these models after studying the nature of their solutions. When finding analytic solutions to these PDEs proved difficult to come by, the authors became interested in semi-analytic and numerical solutions. Numerous semi-analytic techniques, such as Adomian decomposition method (ADM), homotopy analysis method (HAM), and homotopy perturbation method, have been utilized to produce series solutions, however convergence of the series has been a challenge in these solutions which was solved by many semi-analytic techniques like Adomian decomposition method (ADM), homotopy analysis method (HAM), homotopy perturbation method (HPM) and modified variational iteration method (MVIM) [2, 3].

Furthermore, these techniques have successfully handled a variety of linear and nonlinear events. Many authors focused on linearization for nonlinear problems, which did not change the real phenomena but did increase the computational cost. The authors have always contributed to the development of useful methodologies for solving linear and nonlinear PDEs analytically, like tanh-sech method, sine-cosine method, inverse scattering technique, F-expansion technique, consistent Ricatti expansion technique, tanh-function procedure, Hirota’s bilinear scheme, exp-function technique and Jacobi elliptic functions’ scheme and so on [4–7].

The Kortweg-de Vries equation is concerned with shallow water travelling waves. In plasma, nonlinear optics, and hydrodynamics, the idea of travelling waves is used. Pomeau *et al*. [8] used the term seventh-order KdV in a research study to characterize the stability of the equation rediscovered by Kortweg and de Vries under a unique perturbation. Furthermore, infinite many conservation laws (CL) have been determined for seventh-order and ninth-order KdV equations. There has been tremendous growth in the effort to improve solutions of nonlinear KdV equations of orders three and five [9]. The literature for the solutions of KdV equations of orders seven and nine is limited.

Ablowitz brought up the inverse scattering transform and Malfliet explored tanh-technique to deal with the nonlinear phenomena of physical significance. Authors acquired the soliton solutions and the rational solutions of the nonlinear evolution equations [10]. Wazwaz [1] explored the new ninth-order nonlinear dispersive KdV equation and the sixth-order Boussinesq equation. This scheme depended on the generalization of bilinear forms of these equations. The author set out to use the Tanh coth technique for single soliton solutions and a hybrid of the Hirota and Hereman methods [11–13], to show that these equations are fully integrable [1, 14].

However, fast and accurate numerical schemes (finite element method (FEM), finite difference method (FDM) and finite volume method (FVM), spectral and mesh free are used to approximate the DE and IE. Along with many advantages, these methods are observed with a few limitations. To obtain better numerical schemes, researchers have struggled with computational techniques (including wavelet techniques) [15, 16].

Mallat examined the theory of multiresolution analysis (MRA) in order to discuss higher-order resolution for singular and non-linear phenomena [17]. Morlet and Grossmann invented the word wavelet [18]. In 1988, Daubechies introduced a wavelet technique with scaling functions and compact support [17]. Wavelet techniques made it easier for the user by providing good performance with singularities, approximate designs, and ambiguous development. Wavelets are basically depicted on Galerkin techniques and collocation method, in particular, Haar wavelet (HW) is considered simple among different wavelets in the literature. A Hungarian mathematician Alfred Haar established the framework of Haar wavelet [19].

The Haar wavelet contributes to the functions that are piecewise constant and have a simple orthonormal base and local support, the main reason for preferring the Haar wavelet over other wavelets. In addition, at points of discontinuity where its derivatives do not exist, the concept of integration is introduced in order to overcome the prescribed problem [20]. The Haar wavelet techniques have been used for various purposes, for example to eliminate noise from images and signals, for time-frequency analysis, to solve linear and non-linear integro differential equations (IDE), DE and IE [21–29]. Siraj *et al*. investigated a multi-resolution collocation method for time-dependent inverse heat problems [30], while Aziz and Siraj developed a new method to address the two-dimensional elliptical PDEs with HW [31]. Kaya *et al*. solved the fractional equation for KdV Burgers Kuramato using HW scheme [25].

The Kortweg de Vries (KdV) equation of order nine is given by [1]:

$$\begin{array}{c} \begin{array}{cc} & \varpi_{t}+45\varpi_{x}\varpi_{6x}+45\varpi \varpi_{7x}+210\varpi_{3x}\varpi_{4x}+210\varpi_{2x}\varpi_{5x}+1575\varpi_{x}\varpi_{2x}^{2}+3150\varpi \varpi_{2x}\varpi_{3x}\\ & +1260\varpi \varpi_{x}\varpi_{4x}+630\varpi^{2}\varpi_{5x}+9450\varpi^{2}\varpi_{x}\varpi_{2x}+3150\varpi^{3}\varpi_{3x}+4725\varpi^{4}\varpi_{x}+\varpi_{9x}=0, \end{array} \end{array}$$ (1)

depending upon the initial condition(IC):

$$\begin{array}{c} \varpi \left(x,0\right)=\ddot{f}\left(x\right), \end{array}$$ (2)

and the boundary conditions(BCs):

$$\begin{array}{c} \begin{array}{cc} & \varpi \left(0,t\right)={\ddot{\zeta }}_{1}\left(t\right),\;\varpi \left(1,t\right)={\ddot{\zeta }}_{2}\left(t\right),\;\varpi_{x}\left(0,t\right)={\ddot{\zeta }}_{3}\left(t\right),\;\varpi_{x}\left(1,t\right)={\ddot{\zeta }}_{4}\left(t\right),\varpi_{2x}\left(0,t\right)={\ddot{\zeta }}_{5}\left(t\right),\\ & \varpi_{2x}\left(1,t\right)={\ddot{\zeta }}_{6}\left(t\right),\;\varpi_{3x}\left(0,t\right)={\ddot{\zeta }}_{7}\left(t\right),\;\varpi_{3x}\left(1,t\right)={\ddot{\zeta }}_{8}\left(t\right),\;\varpi_{4x}\left(0,t\right)={\ddot{\zeta }}_{9}\left(t\right). \end{array} \end{array}$$ (3)

where ϖ(x,t) is an unknown function of two variables *x* (space variable) and *t* (temporal variable), the domain is denoted by $\overset{ˇ}{\Omega }$, whereas the boundary is expressed by $\partial \overset{ˇ}{\Omega }$. The term $\varpi_{9x}$ is linear and the terms $45\varpi_{x}\varpi_{6x}$, $45\varpi \varpi_{7x}$, $210\varpi_{3x}\varpi_{4x}$, $210\varpi_{2x}\varpi_{5x}$, $1575\varpi_{x}\varpi_{2x}^{2}$, $3150\varpi \varpi_{2x}\varpi_{3x}$, $1260\varpi \varpi_{x}\varpi_{4x}$, $630\varpi^{2}\varpi_{5x}$, $9450\varpi^{2}\varpi_{x}\varpi_{2x}$, $3150\varpi^{3}\varpi_{3x}$ and $4725\varpi^{4}\varpi_{x}$ are nonlinear in Eq (1).

The major objective of this article is to propose a robust numerical technique, to approximate nonlinear KdV equation of order nine, that yields computed results in a small number of collocation points(CPs).

The proposed method in this article is a part of Ph.D. thesis [32]. It is organized in the following manner:

Section 2 explains the topics of multi-resolution analysis and functions of Haar wavelet. Section 3 prescribes the proposed numerical scheme for KdV equation of order nine. Section 4 shows the convergence of the proposed method. A numerical example and justifications are given in Section 5. Finally, in Section 6, some conclusions are drawn from the proposed research work.

## 2 Materials and methods

### 2.1 Multi-resolution analysis

The better understanding of wavelet functions can be achieved by multi-resolution analysis (MRA, a sequence of spaces {${\ddot{Ϝ}}_{j}$}), with the following properties:

1. ${\ddot{\text{Ϝ}}}_{j}\subset {\ddot{\text{Ϝ}}}_{j+1}$
2. ${⋃}_{j\in Z}{\ddot{\text{Ϝ}}}_{j}=L_{2}\left(R\right)$
3. ${⋂}_{j\in Z}{\ddot{\text{Ϝ}}}_{j}=\left\{0\right\}$
4. If $\ddot{f}\left(x\right)\in {\ddot{\text{Ϝ}}}_{j}$, then $\ddot{f}\left(2x\right)\in {\ddot{\text{Ϝ}}}_{j+1}$
5. If $\ddot{f}\left(x\right)\in {\ddot{\text{Ϝ}}}_{j}$, then $\ddot{f}\left(x-k\right)\in {\ddot{\text{Ϝ}}}_{j}$
6. The set of functions $\left\{{\ddot{\phi }}_{j,k}\right\}=2^{j/2}\left(2^{j}x-k\right)$ describes a basis in ${\ddot{\text{Ϝ}}}_{j}$

where $\ddot{f}$ is a square integrable function over the real line and *L*_2_(*R*) is a function space, if the space ${\ddot{\text{Ϝ}}}_{j}$ is defined as:

$$\begin{array}{c} {\ddot{\text{Ϝ}}}_{j}={\ddot{\Lambda }}_{j-1}⊕{\ddot{\text{Ϝ}}}_{j-1}={\ddot{\Lambda }}_{j-1}⊕{\ddot{\Lambda }}_{j-2}⊕{\ddot{\text{Ϝ}}}_{j-2}=\overset{j+1}{\underset{j=1}{⊕}}{\ddot{\Lambda }}_{j}⊕{\ddot{\Lambda }}_{0}. \end{array}$$ (4)

then after scaling and translation, the analysis of multi-resolution(MR) can be constructed for $\left\{{\ddot{\text{Ϝ}}}_{j},j\in Z\right\}$ (the sequence of spaces) that are described by Eqs (5) and (7) on using ${\ddot{h}}_{1}\left(x\right)$. Therefore, ${\ddot{\text{Ϝ}}}_{j+1}\subset {\ddot{\Lambda }}_{j}$, that are ⊥ to ${\ddot{\text{Ϝ}}}_{j},\;\forall \;j$ [17].

### 2.2 Haar wavelet functions

The Haar wavelet (HW) functions are basically step functions with bounded intervals. Here the interval of interest is [0, 1] for all the problems. The HW functions are visualized by different resolution levels. By increasing level of resolution, the better approximation can be achieved using these functions, defined on the interval [0, 1] except father wavelet. The HW functions are with the unique representation given as below [20]:

$$\begin{array}{c} {\ddot{h}}_{i}\left(x\right)=\{\begin{array}{cc} 1, & \forall \;x\in [\pi ,\rho )\\ -1, & \forall \;x\in [\rho ,\sigma )\;\;\&\;\;i=2,3,\ldots \\ 0, & \text{elsewhere} \end{array} \end{array}$$ (5)

where

$$\begin{array}{c} \pi =\frac{k}{o},\;\rho =\frac{k+0.5}{o},\;\sigma =\frac{k+1}{o}, \end{array}$$ (6)

where *π*, *ρ* and *σ* are constants and *j* indicates the HW level, *o* is resolution level where *o* = 2^*j*^, *j* = 0, 1, …, *J* with *J* is the maximum resolution level, is the translation parameter and the index *i* is used for wavelet number.

The father wavelet has the following representation:

$$\begin{array}{c} {\ddot{h}}_{1}\left(x\right)=\{\begin{array}{cc} 1, & \text{for}\;x\in [0,1)\\ 0, & \text{elsewhere} \end{array} \end{array}$$ (7)

All the members of square integrable functions’ family described on the interval [0, 1] can have the following representation, that is in the form of summation of members of HW family:

$$\begin{array}{c} \ddot{f}\left(x\right)=\sum_{i=1}^{\infty }a_{i}{\ddot{h}}_{i}\left(x\right). \end{array}$$ (8)

We identify Θ = 2^*J*^ and Γ = 2Θ, where *J* is defined before. The summation of HW functions

$$\begin{array}{c} \ddot{f}\left(x\right)=\sum_{i=1}^{\Gamma }a_{i}{\ddot{h}}_{i}\left(x\right). \end{array}$$ (9)

is an approximation of $\ddot{f}\left(x\right)$ defined on the interval [0, 1).

The characteristics for integrals of Haar functions are introduced by

$${\ddot{\kappa }}_{i,1}\left(x\right)=\int_{0}^{x}{\ddot{h}}_{i}\left(x^{\prime }\right)dx^{\prime },$$

$${\ddot{\kappa }}_{i,\upsilon +1}\left(x\right)=\int_{0}^{x}{\ddot{\kappa }}_{i,\upsilon }\left(x^{\prime }\right)dx^{\prime },\;\;\;\;\upsilon =1,2,\ldots$$

From Eq (5), the first two integrals may be calculated as:

$$\begin{array}{c} {\ddot{\kappa }}_{i,1}\left(x\right)=\{\begin{array}{cc} x-\pi , & \text{for}\;x\in [\pi ,\rho )\\ \sigma -x, & \text{for}\;x\in [\rho ,\sigma )\\ 0, & otherwise. \end{array} \end{array}$$ (10)

$$\begin{array}{c} {\ddot{\kappa }}_{i,2}\left(x\right)=\{\begin{array}{cc} \frac{1}{2}{\left(x-\pi \right)}^{2}, & \text{for}\;x\in [\pi ,\rho )\\ \frac{1}{2}{\left(\sigma -x\right)}^{2}, & \text{for}\;x\in [\rho ,\sigma )\\ 0, & otherwise \end{array} \end{array}$$ (11)

Generally, the interpretation for the Haar integrals(HI) is given by

$$\begin{array}{c} {\ddot{\kappa }}_{i,\tau }\left(x\right)=\{\begin{array}{cc} 0, & \text{for}\;x\in [0,\pi )\\ \frac{1}{\tau !}{\left(x-\pi \right)}^{\tau }, & \text{for}\;x\in [\pi ,\rho )\\ \frac{1}{\tau !}[{\left(x-\pi \right)}^{\tau }-2{\left(x-\rho \right)}^{\tau }], & \text{for}\;x\in [\rho ,\sigma )\\ \frac{1}{\tau !}[{\left(x-\pi \right)}^{\tau }-2{\left(x-\rho \right)}^{\tau }+{\left(x-\sigma \right)}^{\tau }], & \text{for}\;x\in [\sigma ,1),\tau =1,2,3,\ldots \end{array} \end{array}$$ (12)

where *i* = 2, 3, … and for *i* = 1, we have

$$\begin{array}{c} {\ddot{\kappa }}_{i,\tau }\left(x\right)=\frac{x^{\tau }}{\tau !},\;\;\;\;\;\text{for}\;\tau =1,2,\ldots \end{array}$$

## 3 The proposed approximation method

The proposed method, based on finite difference method and one-dimensional Haar wavelet collocation method (HWCM) is discussed. In Eq (1), the derivatives w.r.t. space variable *x* are discretized using one-dimensional HW formula, while the derivative w.r.t. time variable *t* is estimated by finite difference method as:

$$\begin{array}{c} \frac{\partial \varpi }{\partial t}\approx \frac{\varpi^{\tau +1}-\varpi^{\tau }}{\Delta t}, \end{array}$$ (13)

where *ϖ^τ^* = *ϖ*(*x*, *t_τ_*), *t*_*τ*+1_ = *t*_*τ*_ + Δ*t*, *τ* = 0, 1, …, ⊺/Δ*t* with *t*_0_ = 0. Assuming $x\in \overset{ˇ}{\Omega }=\left[0,1\right]$ and *t* > 0, the following CPs are taken into account:

$$\begin{array}{c} x_{l}=\frac{l-0.5}{2\Theta },\;l=1,2,\ldots 2\Theta . \end{array}$$ (14)

$$\begin{array}{c} t_{l}=\frac{l-0.5}{2\Theta },\;l=1,2,\ldots 2\Theta . \end{array}$$ (15)

Applying FDM to Eq (1)

$$\begin{array}{c} \begin{array}{cc} & \frac{\varpi^{\tau +1}-\varpi^{\tau }}{\Delta t}+45\varpi_{x}^{\tau +1}\varpi_{6x}^{\tau +1}+45\varpi^{\tau +1}\varpi_{7x}^{\tau +1}+210\varpi_{3x}^{\tau +1}\varpi_{4x}^{\tau +1}+210\varpi_{2x}^{\tau +1}\varpi_{5x}^{\tau +1}+1575\varpi_{x}^{\tau +1}\varpi_{2x}^{2(\tau +1)}\\ & +\varpi_{9x}^{\tau +1}+3150\varpi^{\tau +1}\varpi_{2x}^{\tau +1}\varpi_{3x}^{\tau +1}+1260\varpi^{\tau +1}\varpi_{x}^{\tau +1}\varpi_{4x}^{\tau +1}+630\varpi^{2(\tau +1)}\varpi_{5x}^{\tau +1}+9450\varpi^{2(\tau +1)}\varpi_{x}^{\tau +1}\varpi_{2x}^{\tau +1}\\ & +3150\varpi^{3(\tau +1)}\varpi_{3x}^{\tau +1}+4725\varpi^{4(\tau +1)}\varpi_{x}^{\tau +1}=0. \end{array} \end{array}$$ (16)

After doing linearization of the nonlinear terms of Eq (16)

$$\begin{array}{c} \begin{array}{cc} & (1+\Delta t(45\varpi_{7x}^{\tau }+3150\varpi_{3x}^{\tau }\varpi_{2x}^{\tau }+1260\varpi_{x}^{\tau }\varpi_{4x}^{\tau }+1260\varpi^{\tau }\varpi_{5x}^{\tau }+18900\varpi^{\tau }u_{x}^{\tau }\varpi_{2x}^{\tau }+9450\varpi^{2\tau }\varpi_{3x}^{\tau }\\ & +18900\varpi^{3\tau }\varpi_{x}^{n}))\varpi^{\tau +1}+\Delta t(45\varpi_{6x}^{\tau }+1575\varpi_{2x}^{2\tau }+1260\varpi^{\tau }\varpi_{4x}^{\tau }+9450\varpi^{2\tau }\varpi_{2x}^{\tau }+4725\varpi^{4\tau })\varpi_{x}^{\tau +1}\\ & +\Delta t(210\varpi_{5x}^{\tau }+3150\varpi^{\tau }\varpi_{3x}^{\tau }+3150\varpi_{x}^{\tau }\varpi_{2x}^{\tau }+9450\varpi^{2\tau }\varpi_{x}^{\tau })\varpi_{2x}^{\tau +1}\\ & +\Delta t(210\varpi_{4x}^{\tau }+3150\varpi^{\tau }\varpi_{2x}^{\tau }+3150\varpi^{3\tau })\varpi_{3x}^{\tau +1}+\Delta t(1260\varpi^{\tau }\varpi_{x}^{\tau }+210\varpi_{3x}^{\tau })\varpi_{4x}^{\tau +1}\\ & +\Delta t(210\varpi_{2x}^{\tau }+630\varpi^{2\tau })\varpi_{5x}^{\tau +1}+45\Delta t\varpi_{x}^{\tau }\varpi_{6x}^{\tau +1}+45\Delta t\varpi^{\tau }\varpi_{7x}^{\tau +1}+\Delta t\varpi_{9x}^{\tau +1}\\ & =\varpi^{\tau }+45\Delta t\varpi_{x}^{\tau }\varpi_{6x}^{\tau }+45\Delta t\varpi^{\tau }\varpi_{7x}^{\tau }+210\Delta t\varpi_{3x}^{\tau }\varpi_{4x}^{\tau }+420\Delta t\varpi_{2x}^{\tau }\varpi_{5x}^{\tau }+3150\Delta t\varpi_{x}^{\tau }\varpi_{2x}^{\tau }\\ & +6300\Delta t\varpi^{\tau }\varpi_{2x}^{\tau }\varpi_{3x}^{\tau }+2520\Delta t\varpi^{\tau }\varpi_{x}^{\tau }\varpi_{4x}^{\tau }+1260\Delta t\varpi^{2\tau }\varpi_{5x}^{\tau }+28350\Delta t\varpi^{2\tau }\varpi_{x}^{\tau }\varpi_{2x}^{\tau }\\ & +9450\Delta t\varpi^{3\tau }\varpi_{3x}^{\tau }+18900\Delta t\varpi^{4\tau }\varpi_{x}^{\tau }, \end{array} \end{array}$$ (17)

where nonlinear terms: $\varpi_{x}^{\tau +1}\varpi_{6x}^{\tau +1}$, $\varpi^{\tau +1}\varpi_{7x}^{\tau +1}$, $\varpi_{3x}^{\tau +1}\varpi_{4x}^{\tau +1}$, $\varpi_{2x}^{\tau +1}\varpi_{5x}^{\tau +1}$, $\varpi_{x}^{\tau +1}\varpi_{2x}^{2(\tau +1)}$, $\varpi^{2(\tau +1)}\varpi_{5x}^{\tau +1}$, $\varpi^{\tau +1}\varpi_{2x}^{\tau +1}\varpi_{3x}^{\tau +1}$, $\varpi^{\tau +1}\varpi_{x}^{\tau +1}\varpi_{4x}^{\tau +1}$, $\varpi^{2(\tau +1)}\varpi_{x}^{\tau +1}\varpi_{2x}^{\tau +1}$, $\varpi^{3(\tau +1)}\varpi_{3x}^{\tau +1}$ and $\varpi^{4(\tau +1)}\varpi_{x}^{\tau +1}$ are linearized by quasi-Newton linearization technique:

$$\begin{array}{c} \varpi_{x}^{\tau +1}\varpi_{6x}^{\tau +1}=\varpi_{x}^{\tau }\varpi_{6x}^{\tau +1}+\varpi_{x}^{\tau +1}\varpi_{6x}^{\tau }-\varpi_{x}^{\tau }\varpi_{6x}^{\tau }, \end{array}$$

$$\begin{array}{c} \varpi^{\tau +1}\varpi_{7x}^{\tau +1}=\varpi^{\tau }\varpi_{7x}^{\tau +1}+\varpi^{\tau +1}\varpi_{7x}^{\tau }-\varpi^{\tau }\varpi_{7x}^{\tau }, \end{array}$$

$$\begin{array}{c} \varpi_{3x}^{\tau +1}\varpi_{4x}^{\tau +1}=\varpi_{3x}^{\tau }\varpi_{4x}^{\tau +1}+\varpi_{3x}^{\tau +1}\varpi_{4x}^{\tau }-\varpi_{3x}^{\tau }\varpi_{4x}^{\tau }, \end{array}$$

$$\begin{array}{c} \varpi_{2x}^{\tau +1}\varpi_{5x}^{\tau +1}=\varpi_{2x}^{\tau }\varpi_{5x}^{\tau +1}+\varpi_{2x}^{\tau +1}\varpi_{5x}^{\tau }-\varpi_{2x}^{\tau }\varpi_{5x}^{\tau }, \end{array}$$

$$\begin{array}{c} \varpi_{x}^{\tau +1}\varpi_{2x}^{2(\tau +1)}=2\varpi_{2x}^{\tau +1}\varpi_{2x}^{\tau }\varpi_{x}^{\tau }+\varpi_{x}^{\tau +1}\varpi_{2x}^{2\tau }-2\varpi_{x}^{\tau }\varpi_{2x}^{2\tau }, \end{array}$$

$$\begin{array}{c} \varpi^{3(\tau +1)}\varpi_{3x}^{\tau +1}=\varpi^{3\tau }\varpi_{3x}^{\tau +1}+3\varpi^{2\tau }\varpi^{\tau +1}\varpi_{3x}^{\tau }-3\varpi^{3\tau }\varpi_{3x}^{\tau }, \end{array}$$

$$\begin{array}{c} \varpi^{4(\tau +1)}\varpi_{x}^{\tau +1}=\varpi^{4\tau }\varpi_{x}^{\tau +1}+4\varpi^{3\tau }u^{\tau +1}\varpi_{x}^{\tau }-4\varpi^{4\tau }\varpi_{x}^{\tau }, \end{array}$$

$$\begin{array}{c} \varpi^{2(\tau +1)}\varpi_{5x}^{\tau +1}=\varpi^{2\tau }\varpi_{5x}^{\tau +1}+2\varpi^{\tau }\varpi^{\tau +1}\varpi_{5x}^{\tau }-2\varpi^{2\tau }\varpi_{5x}^{\tau }, \end{array}$$

$$\begin{array}{c} \varpi^{\tau +1}\varpi_{2x}^{\tau +1}\varpi_{3x}^{\tau +1}=\varpi^{\tau }\varpi_{2x}^{\tau }\varpi_{3x}^{\tau +1}+\varpi^{\tau }\varpi_{2x}^{\tau +1}\varpi_{3x}^{\tau }+\varpi^{\tau +1}\varpi_{2x}^{\tau }\varpi_{3x}^{\tau }-2\varpi^{\tau }\varpi_{2x}^{\tau }\varpi_{3x}^{\tau }, \end{array}$$

$$\begin{array}{c} \varpi^{\tau +1}\varpi_{x}^{\tau +1}\varpi_{4x}^{\tau +1}=\varpi^{\tau }\varpi_{x}^{\tau }\varpi_{4x}^{\tau +1}+\varpi^{\tau }\varpi_{x}^{\tau +1}\varpi_{4x}^{\tau }+\varpi^{\tau +1}\varpi_{x}^{\tau }\varpi_{4x}^{\tau }-2\varpi^{\tau }\varpi_{x}^{\tau }\varpi_{4x}^{\tau }, \end{array}$$

$$\begin{array}{c} \varpi^{2(\tau +1)}\varpi_{x}^{\tau +1}\varpi_{2x}^{\tau +1}=\varpi^{2\tau }\varpi_{x}^{\tau }\varpi_{2x}^{\tau +1}+2\varpi^{\tau }\varpi_{x}^{\tau }\varpi^{\tau +1}\varpi_{2x}^{\tau }-3\varpi^{2\tau }\varpi_{x}^{\tau }\varpi_{2x}^{\tau }+\varpi^{2\tau }\varpi_{2x}^{\tau }\varpi_{x}^{\tau +1}. \end{array}$$

On substituting CPs

$$\begin{array}{c} \begin{array}{cc} & \left(1+\Delta t(45\varpi_{7x_{l}}^{\tau }+3150\varpi_{3x_{l}}^{\tau }\varpi_{2x_{l}}^{\tau }+1260\varpi_{x_{l}}^{\tau }\varpi_{4x_{l}}^{\tau }+1260\varpi^{\tau }\varpi_{5x_{l}}^{\tau }+18900\varpi^{\tau }u_{x_{l}}^{\tau }\varpi_{2x_{l}}^{\tau }+9450\varpi^{2\tau }\varpi_{3x_{l}}^{\tau }+18900\varpi^{3\tau }\varpi_{x_{l}}^{\tau })\right)\\ & \varpi^{\tau +1}+\Delta t(45\varpi_{6x_{l}}^{\tau }+1575\varpi_{2x_{l}}^{2\tau }+1260\varpi^{\tau }\varpi_{4x_{l}}^{\tau }+9450\varpi^{2\tau }\varpi_{2x_{l}}^{\tau }+4725\varpi^{4\tau })\varpi_{x_{l}}^{\tau +1}+\Delta t(210\varpi_{2x_{l}}^{\tau }+630\varpi^{2\tau })\varpi_{5x_{l}}^{\tau +1}\\ & +\Delta t(210\varpi_{4x_{l}}^{\tau }+3150\varpi^{\tau }\varpi_{2x_{l}}^{\tau }+3150\varpi^{3\tau })\varpi_{3x_{l}}^{\tau +1}+\Delta t(1260\varpi^{\tau }\varpi_{x_{l}}^{\tau }+210\varpi_{3x_{l}}^{\tau })\varpi_{4x_{l}}^{\tau +1}++45\Delta t\varpi_{x_{l}}^{\tau }\varpi_{6x_{l}}^{\tau +1}+\Delta t\varpi_{9x_{l}}^{\tau +1}\\ & +45\Delta t\varpi^{\tau }\varpi_{7x_{l}}^{\tau +1}+\Delta t(210\varpi_{5x_{l}}^{\tau }+3150\varpi^{\tau }\varpi_{3x_{l}}^{\tau }+3150\varpi_{x_{l}}^{\tau }\varpi_{2x_{l}}^{\tau }+9450\varpi^{2\tau }\varpi_{x_{l}}^{\tau })\varpi_{2x_{l}}^{\tau +1}=\varpi^{\tau }+45\Delta t\varpi_{x_{l}}^{\tau }\varpi_{6x_{l}}^{\tau }+45\Delta t\\ & \varpi^{\tau }\varpi_{7x_{l}}^{\tau }+210\Delta t\varpi_{3x_{l}}^{\tau }\varpi_{4x_{l}}^{\tau }+420\Delta t\varpi_{2x_{l}}^{\tau }\varpi_{5x_{l}}^{\tau }+3150\Delta t\varpi_{x_{l}}^{\tau }\varpi_{2x_{l}}^{\tau }+6300\Delta t\varpi^{\tau }\varpi_{2x_{l}}^{\tau }\varpi_{3x_{l}}^{\tau }+2520\Delta t\varpi^{\tau }\varpi_{x_{l}}^{\tau }\varpi_{4x_{l}}^{\tau }+1260\Delta t\\ & \varpi^{2\tau }\varpi_{5x_{l}}^{\tau }+28350\Delta t\varpi^{2\tau }\varpi_{x_{l}}^{\tau }\varpi_{2x_{l}}^{\tau }+9450\Delta t\varpi^{3\tau }\varpi_{3x_{l}}^{\tau }+18900\Delta t\varpi^{4\tau }\varpi_{x_{l}}^{\tau }, \end{array} \end{array}$$ (18)

where *l* = 1, 2, …, 2Θ are CPs.

The estimation of highest derivative of KdV equation in Eq (1) is done by HW functions as:

$$\begin{array}{c} \varpi_{9x}\left(x,t\right)=\sum_{i=1}^{\Gamma }a_{i}{\ddot{h}}_{i}\left(x\right). \end{array}$$ (19)

The following expressions can be obtained by integrating Eq (19) as:

$$\begin{array}{c} \varpi_{8x}\left(x,t\right)=\varpi_{8x}\left(0,t\right)+\sum_{i=1}^{\Gamma }a_{i}{\ddot{\kappa }}_{i,1}\left(x\right). \end{array}$$ (20)

$$\begin{array}{c} \varpi_{7x}\left(x,t\right)=\varpi_{7x}\left(0,t\right)+x\varpi_{8x}\left(0,t\right)+\sum_{i=1}^{\Gamma }a_{i}{\ddot{\kappa }}_{i,2}\left(x\right). \end{array}$$ (21)

$$\begin{array}{c} \varpi_{6x}\left(x,t\right)=\varpi_{6x}\left(0,t\right)+x\varpi_{7x}\left(0,t\right)+\frac{x^{2}}{2}\varpi_{8x}\left(0,t\right)+\sum_{i=1}^{\Gamma }a_{i}{\ddot{\kappa }}_{i,3}\left(x\right). \end{array}$$ (22)

$$\begin{array}{c} \begin{array}{c} \varpi_{5x}\left(x,t\right)=\varpi_{5x}\left(0,t\right)+x\varpi_{6x}\left(0,t\right)+\frac{x^{2}}{2}\varpi_{7x}\left(0,t\right)+\frac{x^{3}}{6}\varpi_{8x}\left(0,t\right)+\sum_{i=1}^{\Gamma }a_{i}{\ddot{\kappa }}_{i,4}\left(x\right). \end{array} \end{array}$$ (23)

$$\begin{array}{c} \begin{array}{c} \varpi_{4x}\left(x,t\right)=\varpi_{4x}\left(0,t\right)+x\varpi_{5x}\left(0,t\right)+\frac{x^{2}}{2}\varpi_{6x}\left(0,t\right)+\frac{x^{3}}{6}\varpi_{7x}\left(0,t\right)+\frac{x^{4}}{24}\varpi_{8x}\left(0,t\right)+\sum_{i=1}^{\Gamma }a_{i}{\ddot{\kappa }}_{i,5}\left(x\right). \end{array} \end{array}$$ (24)

$$\begin{array}{c} \begin{array}{cc} & \varpi_{3x}\left(x,t\right)=\varpi_{3x}\left(0,t\right)+x\varpi_{4x}\left(0,t\right)+\frac{x^{2}}{2}\varpi_{5x}\left(0,t\right)+\frac{x^{3}}{6}\varpi_{6x}\left(0,t\right)+\frac{x^{4}}{24}\varpi_{7x}\left(0,t\right)+\frac{x^{5}}{120}\varpi_{8x}\left(0,t\right)\\ & +\sum_{i=1}^{\Gamma }a_{i}{\ddot{\kappa }}_{i,6}\left(x\right). \end{array} \end{array}$$ (25)

$$\begin{array}{c} \begin{array}{cc} & \varpi_{2x}\left(x,t\right)=\varpi_{2x}\left(0,t\right)+x\varpi_{3x}\left(0,t\right)+\frac{x^{2}}{2}\varpi_{4x}\left(0,t\right)+\frac{x^{3}}{6}\varpi_{5x}\left(0,t\right)+\frac{x^{4}}{24}\varpi_{6x}\left(0,t\right)+\frac{x^{5}}{120}\varpi_{7x}\left(0,t\right)\\ & +\frac{x^{6}}{720}\varpi_{8x}\left(0,t\right)+\sum_{i=1}^{\Gamma }a_{i}{\ddot{\kappa }}_{i,7}\left(x\right). \end{array} \end{array}$$ (26)

$$\begin{array}{c} \begin{array}{cc} & \varpi_{x}\left(x,t\right)=\varpi_{x}\left(0,t\right)+x\varpi_{2x}\left(0,t\right)+\frac{x^{2}}{2}\varpi_{3x}\left(0,t\right)+\frac{x^{3}}{6}\varpi_{4x}\left(0,t\right)+\frac{x^{4}}{24}\varpi_{5x}\left(0,t\right)+\frac{x^{5}}{120}\varpi_{6x}\left(0,t\right)\\ & +\frac{x^{6}}{720}\varpi_{7x}\left(0,t\right)+\frac{x^{7}}{5040}\varpi_{8x}\left(0,t\right)+\sum_{i=1}^{\Gamma }a_{i}{\ddot{\kappa }}_{i,8}\left(x\right). \end{array} \end{array}$$ (27)

$$\begin{array}{c} \begin{array}{cc} & \varpi \left(x,t\right)=\varpi \left(0,t\right)+x\varpi_{x}\left(0,t\right)+\frac{x^{2}}{2}\varpi_{2x}\left(0,t\right)+\frac{x^{3}}{6}\varpi_{3x}\left(0,t\right)+\frac{x^{4}}{24}\varpi_{4x}\left(0,t\right)+\frac{x^{5}}{120}\varpi_{5x}\left(0,t\right)\\ & +\frac{x^{6}}{720}\varpi_{6x}\left(0,t\right)+\frac{x^{7}}{5040}\varpi_{7x}\left(0,t\right)+\frac{x^{8}}{40320}\varpi_{8x}\left(0,t\right)+\sum_{i=1}^{\Gamma }a_{i}{\ddot{\kappa }}_{i,9}\left(x\right). \end{array} \end{array}$$ (28)

The unknown values $\varpi_{5x}\left(0,t\right)$, $\varpi_{6x}\left(0,t\right)$, $\varpi_{7x}\left(0,t\right)$ and $\varpi_{8x}\left(0,t\right)$ are computed as:

$$\begin{array}{c} \begin{array}{cc} & \varpi_{5x}\left(0,t\right)=-6720\varpi \left(0,t\right)+6720\varpi \left(1,t\right)-4200\varpi_{x}\left(0,t\right)-2520\varpi_{x}\left(1,t\right)-1200\varpi_{2x}\left(0,t\right)+360\varpi_{2x}\left(1,t\right)\\ & -200\varpi_{3x}\left(0,t\right)-20\varpi_{3x}\left(1,t\right)-20\varpi_{4x}\left(0,t\right)+20\sum_{i=1}^{\Gamma }a_{i}{\ddot{\kappa }}_{i,6}\left(1\right)-360\sum_{i=1}^{\Gamma }a_{i}{\ddot{\kappa }}_{i,7}\left(1\right)+2520\sum_{i=1}^{\Gamma }a_{i}{\ddot{\kappa }}_{i,8}\left(1\right)\\ & -6720\sum_{i=1}^{\Gamma }a_{i}{\ddot{\kappa }}_{i,9}\left(1\right). \end{array} \end{array}$$ (29)

$$\begin{array}{c} \begin{array}{cc} & \varpi_{6x}\left(0,t\right)=100800\varpi \left(0,t\right)-100800\varpi \left(1,t\right)+60480\varpi_{x}\left(0,t\right)+40320\varpi_{x}\left(1,t\right)+16200\varpi_{2x}\left(0,t\right)\\ & -6120\varpi_{2x}\left(1,t\right)+2400\varpi_{3x}\left(0,t\right)+360\varpi_{3x}\left(1,t\right)+180\varpi_{4x}\left(0,t\right)-360\sum_{i=1}^{\Gamma }a_{i}{\ddot{\kappa }}_{i,6}\left(1\right)+6120\sum_{i=1}^{\Gamma }a_{i}{\ddot{\kappa }}_{i,7}\left(1\right)\\ & -40320\sum_{i=1}^{\Gamma }a_{i}{\ddot{\kappa }}_{i,8}\left(1\right)+100800\sum_{i=1}^{\Gamma }a_{i}{\ddot{\kappa }}_{i,9}\left(1\right). \end{array} \end{array}$$ (30)

$$\begin{array}{c} \begin{array}{cc} & \varpi_{7x}\left(0,t\right)=-604800\varpi \left(0,t\right)+604800\varpi \left(1,t\right)-352800\varpi_{x}\left(0,t\right)-252000\varpi_{x}\left(1,t\right)-90720\varpi_{2x}\left(0,t\right)\\ & +40320\varpi_{2x}\left(1,t\right)-12600\varpi_{3x}\left(0,t\right)-2520\varpi_{3x}\left(1,t\right)-840\varpi_{4x}\left(0,t\right)+2520\sum_{i=1}^{\Gamma }a_{i}{\ddot{\kappa }}_{i,6}\left(1\right)\\ & -40320\sum_{i=1}^{\Gamma }a_{i}{\ddot{\kappa }}_{i,7}\left(1\right)+252000\sum_{i=1}^{\Gamma }a_{i}{\ddot{\kappa }}_{i,8}\left(1\right)-604800\sum_{i=1}^{\Gamma }a_{i}{\ddot{\kappa }}_{i,9}\left(1\right). \end{array} \end{array}$$ (31)

$$\begin{array}{c} \begin{array}{cc} & \varpi_{8x}\left(0,t\right)=1411200\varpi \left(0,t\right)-1411200\varpi \left(1,t\right)+806400\varpi_{x}\left(0,t\right)+604800\varpi_{x}\left(1,t\right)+201600\varpi_{2x}\left(0,t\right)\\ & -100800\varpi_{2x}\left(1,t\right)+26880\varpi_{3x}\left(0,t\right)+6720\varpi_{3x}\left(1,t\right)+1680\varpi_{4x}\left(0,t\right)-6720\sum_{i=1}^{\Gamma }a_{i}{\ddot{\kappa }}_{i,6}\left(1\right)\\ & +100800\sum_{i=1}^{\Gamma }a_{i}{\ddot{\kappa }}_{i,7}\left(1\right)-604800\sum_{i=1}^{\Gamma }a_{i}{\ddot{\kappa }}_{i,8}\left(1\right)+1411200\sum_{i=1}^{\Gamma }a_{i}{\ddot{\kappa }}_{i,9}\left(1\right). \end{array} \end{array}$$ (32)

After substituting the above statements in Eq (18), a system of equations is obtained. Next, to find Haar coefficients(HCs) for the solution at time *t*_*τ*+1_, the known values of $\varpi_{l}$’s at time *t*_*τ*_ are replaced in the obtained system of equations. Next, the actual solution of the prescribed problem is obtained by these HCs. Because of iterative scheme, we can obtain the solution at any particular time.

## 4 Convergence of the proposed method [33]

**Theorem 1** *Suppose that a function* $\ddot{f}\left(x\right)=\frac{d^{\tau }\varpi \left(x\right)}{dx^{\tau }}\in L^{2}\left(R\right)$ *is continuous on the closed interval* [0, 1], *with its restricted first order derivative then for all x belonging to the interval [0, 1], there exists η, such that*:

$$\begin{array}{c} |\frac{d\ddot{f}\left(x\right)}{dx}|\leq \eta ,\;\tau \geq 2. \end{array}$$

*Later, the HW technique, constructed on the basis of the scheme that is proposed in* [34] *converges, i.e*. $|{\tilde{E}}_{o}|$ *evaporates when J* → ∞, *the order convergence is 2*.

$$\begin{array}{c} \|{\tilde{E}}_{o}{\|}_{2}=O\left[{\left(\frac{1}{2^{J+1}}\right)}^{2}\right], \end{array}$$

*where*

$$\begin{array}{c} {\tilde{E}}_{o}=\ddot{f}\left(x\right)-{\ddot{f}}_{\Theta }\left(x\right),\;{\ddot{f}}_{\Theta }\left(x\right)=\sum_{i=0}^{\Gamma }a_{i}{\ddot{h}}_{i}\left(x\right). \end{array}$$

## 5 Results

To examine the strategy of the proposed technique, a test problem is considered. The interval for the proposed test problem is taken [0, 1]. The symbol *E*_*c*_(Γ) is used to determine maximum absolute errors (MAEs). The notation *R*_*c*_(Γ) will be used to denote the experimental rate of convergence with Γ = 2Θ number of collocation points.

The experimental rate of convergence is defined as follows:

$$\begin{array}{c} R_{c}\left(\Gamma \right)=\frac{\text{log}\;(E_{c}\left(\Gamma /2\right)/E_{c}\left(\Gamma \right))}{\text{log}\;2}. \end{array}$$ (33)

**Test Problem 1** *Consider the equation of Kortweg-de Vries having order nine, given in* Eq (1) *with the series solution* [2]:

$$\begin{array}{c} \begin{array}{cc} & \varpi \left(x,t\right)=\frac{2b{\overset{ˇ}{k}}^{2}(692-273tanh{\left(\sqrt{b}\overset{ˇ}{k}x\right)}^{2})}{273}+\frac{\left(34796911758272i\sqrt[{11}]{b}{\overset{ˇ}{k}}^{11}tsech{\left(\sqrt{b}\overset{ˇ}{k}x\right)}^{2}tanh\left(\sqrt{b}\overset{ˇ}{k}x\right)\right)}{9796423}\\ & +\frac{151353133489121021320053248b^{10}{\overset{ˇ}{k}}^{20}t^{2}(-2+cosh(2\sqrt{b}\overset{ˇ}{k}x))sech{\left(\sqrt{b}\overset{ˇ}{k}x\right)}^{4}}{95969903594929}+\\ & \frac{9459570843070063832503328\sqrt[{29}]{b}{\overset{ˇ}{k}}^{29}t^{3}sech{\left(\sqrt{b}\overset{ˇ}{k}x\right)}^{13}}{2820485312655435416901}(576118951475102sinh\left(\sqrt{b}\overset{ˇ}{k}x\right)+\\ & 240565607778742sinh\left(3\sqrt{b}\overset{ˇ}{k}x\right)-96560861247595sinh\left(5\sqrt{b}\right)-50253988942927sinh\left(7\sqrt{b}\overset{ˇ}{k}x\right)\\ & -4308691386269sinh\left(9\sqrt{b}\overset{ˇ}{k}x\right)+543701746223sinh\left(11\sqrt{b}\overset{ˇ}{k}x\right)))+\ldots \end{array} \end{array}$$ (34)

The initial and boundary conditions are obtained from the series solution. The presented nonlinear KdV equation of order nine is a parabolic equation that describes the water waves phenomenon, while its series solution is a hyperbolic function. In physics, distortion in one-dimensional(1-D) rippling is given by the presented equation, that involves shallow water waves, likewise, in the routine work, hyperbolic functions perform a momentous role as well.

**Fig 1:**
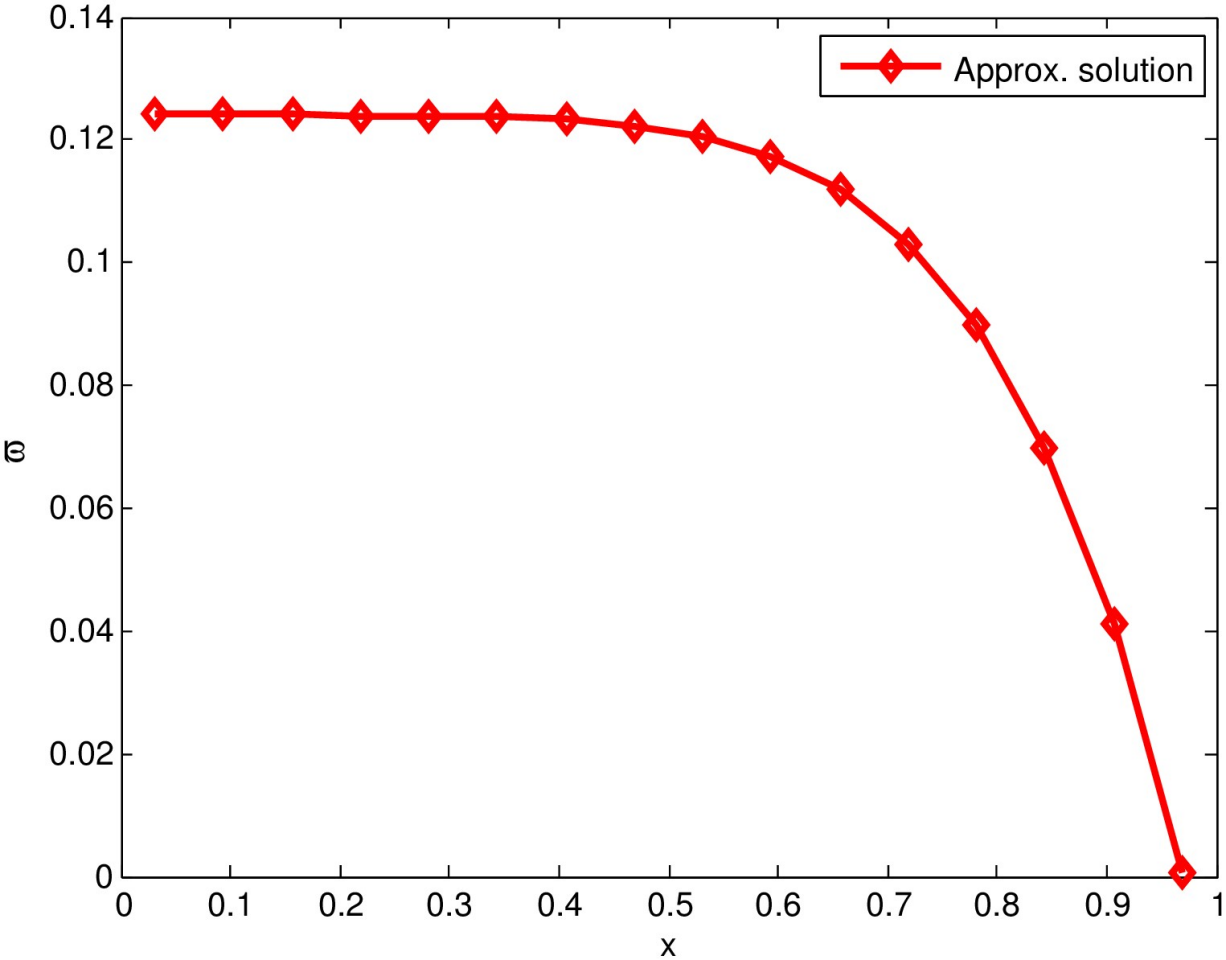


The one-dimensional HWCM is applied on the Eq (1) and Tables 1–3 illustrate its numerical results. The Table 1 represents the point-wise absolute errors (PWAEs) (for *t* = 1, 3, 5, 7, 9). It is noticeable that for *x* = 0, the PWAE is vanished, whereas increase in the other PWAE is observed, if we move along space. The Tables 2 and 3 represent MAEs and the rate of convergence (respectively) at different collocation points for various time step sizes (Δ*t* = 10^−02^, 10^−03^, 10^−04^) and the constant time level *t* = 1. The obtained MAEs are representing the precision of one-dimensional HWCM for different collocation points. The order of PWAEs is 10^−02^ along with Δ*t* = 10^−02^, *b* = 0.2, *t* = 1 and $\overset{ˇ}{k}=0.35$. Moreover, the order of MAEs is 10^−01^ for $\overset{ˇ}{k}=0.35$ and *t* = 1. In the prescribed problem, by lowering the time step size, the progress in the precision of the presented technique is observed, while it (precision) is not increased by increasing the number of collocation points. To fix this problem, some restrictions will be implemented on this procedure. In addition, the Fig 1 indicates the approximate solution of KdV equation of order nine.

**Table 1:** The PWAEs for Γ = 16, Δ*t* = 10^−02^, *b* = 0.2 and $\overset{ˇ}{k}=0.35$ for Test Problem 1.

*x*/*t*	1	3	5	7	9
	PWAE	PWAE	PWAE	PWAE	PWAE
0	0	0	0	0	0
0.1	1.2526 × 10^−07^	9.1128 × 10^−08^	1.7294 × 10^−08^	2.2868 × 10^−08^	1.0987 × 10^−07^
0.2	3.9754 × 10^−06^	4.6259 × 10^−06^	4.0222 × 10^−06^	1.6372 × 10^−06^	5.5316 × 10^−06^
0.3	8.1379 × 10^−05^	7.6963 × 10^−05^	5.8511 × 10^−05^	1.9315 × 10^−05^	5.8555 × 10^−05^
0.4	5.4215 × 10^−04^	4.9241 × 10^−04^	3.5817 × 10^−04^	1.1013 × 10^−04^	3.1918 × 10^−04^
0.5	2.2141 × 10^−03^	1.9831 × 10^−03^	1.4170 × 10^−03^	4.2300 × 10^−04^	1.2011 × 10^−03^
0.6	6.8060 × 10^−03^	6.0625 × 10^−03^	4.2985 × 10^−03^	1.2671 × 10^−03^	3.5656 × 10^−03^
0.7	1.7363 × 10^−02^	1.5435 × 10^−02^	1.0907 × 10^−02^	3.1997 × 10^−03^	8.9758 × 10^−03^
0.8	3.8842 × 10^−02^	3.4508 × 10^−02^	2.4350 × 10^−02^	7.1335 × 10^−03^	2.0010 × 10^−02^
0.9	7.8823 × 10^−02^	7.0039 × 10^−02^	4.9374 × 10^−01^	1.4465 × 10^−02^	4.0636 × 10^−02^
1	1.4836 × 10^−02^	1.3189 × 10^−01^	9.2884 × 10^−02^	2.7218 × 10^−02^	7.6651 × 10^−02^

**Table 2:** The MAEs for $\overset{ˇ}{k}=0.35$ and *t* = 1 for Test Problem 1.

Γ	Δ*t* = 10^−02^	Δ*t* = 10^−03^	Δ*t* = 10^−04^
	*E*_*c*_(Γ)	*E*_*c*_(Γ)	*E*_*c*_Γ
2^0^	2.6323 × 10^−02^	2.6292 × 10^−02^	2.6307 × 10^−02^
2^2^	6.6536 × 10^−02^	6.6535 × 10^−02^	6.6539 × 10^−02^
2^3^	1.0070 × 10^−01^	1.0067 × 10^−01^	1.0067 × 10^−01^
2^4^	1.2262 × 10^−01^	1.2255 × 10^−01^	1.2256 × 10^−01^
2^5^	1.3499 × 10^−01^	1.3490 × 10^−01^	1.3491 × 10^−01^
2^6^	1.4156 × 10^−01^	1.4146 × 10^−01^	1.4146 × 10^−01^
2^7^	1.4493 × 10^−01^	1.4483 × 10^−01^	1.4484 × 10^−01^

**Table 3:** The rate of convergence *R*_*c*_(Γ) for $\overset{ˇ}{k}=0.35$, and *t* = 1 for Test Problem 1.

Γ	Δ*t* = 10^−02^	Δ*t* = 10^−03^	Δ*t* = 10^−04^
	*R*_*c*_(Γ)	*R*_*c*_(Γ)	*R*_*c*_(Γ)
2^0^	—	—	—
2^2^	−1.3378	−1.3395	−1.3388
2^3^	−0.5979	−0.5974	−0.5974
2^4^	−0.2841	−0.2837	−0.2839
2^5^	−0.1387	−0.1385	−0.1385
2^6^	−0.0686	−0.0685	−0.0684
2^7^	−0.0339	−0.0340	−0.0341

## 6 Conclusion

The nonlinear KdV has been approximated by one-dimensional HWCM. The given equation is discretized utilizing finite difference technique and the collocation procedure. The proposed scheme is implemented on KdV equation of order nine and a reasonable performance of the one-dimensional HWCM is observed from the computed results. Furthermore, it is noted, that reducing the size of time step results in an improvement in the precision of the presented technique, while the precision does not increase with increasing collocation points. However, in future, a few constraints are needed to impose on the proposed scheme, to obtain the required increase in precision.
